# Acoustic- and Radio-Frequency-Based Human Activity Recognition

**DOI:** 10.3390/s22093125

**Published:** 2022-04-19

**Authors:** Masoud Mohtadifar, Michael Cheffena, Alireza Pourafzal

**Affiliations:** Faculty of Engineering, Norwegian University of Science and Technology (NTNU), Teknologivegen 22, 2815 Gjøvik, Norway; masoud.mohtadifar@ntnu.no (M.M.); alireza.pourafzal@ntnu.no (A.P.)

**Keywords:** human activity recognition, machine learning, hybrid activity recognition, acoustic-based HAR, RF-based HAR, data fusion

## Abstract

In this work, a hybrid radio frequency (RF)- and acoustic-based activity recognition system was developed to demonstrate the advantage of combining two non-invasive sensors in Human Activity Recognition (HAR) systems and smart assisted living. We used a hybrid approach, employing RF and acoustic signals to recognize falling, walking, sitting on a chair, and standing up from a chair. To our knowledge, this is the first work that attempts to use a mixture of RF and passive acoustic signals for Human Activity Recognition purposes. We conducted experiments in the lab environment using a Vector Network Analyzer measuring the 2.4 GHz frequency band and a microphone array. After recording data, we extracted the Mel-spectrogram feature of the audio data and the Doppler shift feature of the RF measurements. We fed these features to six classification algorithms. Our result shows that using a hybrid acoustic- and radio-based method increases the accuracy of recognition compared to just using only one kind of sensory data and shows the possibility of expanding for a variety of other different activities that can be recognized. We demonstrate that by using a hybrid method, the recognition accuracy increases in all classification algorithms. Among these classifiers, five of them achieve over 98% recognition accuracy.

## 1. Introduction

Smart assisted living has attracted a lot of attention in recent years. This is due to the increasing need for smart living as the population age increases, especially in developed countries [1]. Specifically, falls in elderly people are considered as one of the major causes of severe injuries. This is because most elderly people live alone, and delay in rescue might put their lives in danger.

One solution is to provide Human Activity Recognition (HAR) devices as a smart assisted living system to detect falls. Existing HAR techniques use a wide variety of sensors, such as visual, inertial, acoustic, pressure, etc. HAR using visual techniques has made significant progress and gained high recognition accuracy [2]; however, these techniques raise privacy issues [2]. Furthermore, these visual techniques require hardware installation and high computational power, increasing the cost of deployment [2].

In wearable-sensors-based HAR, the subject puts on a wearable device, usually on their wrist, and the sensors embedded in the device are used to track the subject’s activity and gather data. Based on their embedded sensors, these devices can be used to detect falls, steps, walking speed, heart rate, oxygen level, etc. [2]. However, the challenge with wearable devices is that their operation is limited to just one subject and requires the subject to wear the device, which can cause discomfort and noncompliance, especially in older people who are the target populations for HAR.

Given the shortcomings of the visual and wearable sensor devices, one solution is to employ less intrusive sensors with cheaper deployment costs, such as Device-Free Radio-Based Activity Recognition (DFRAR) devices and acoustic sensors.

In DFRAR, the received signal is affected by the human activities in the channel. As a result of these activities, the received signal may exhibit distinct features that can be exploited for HAR purposes. Wang et al. [3] showed that when human activity is performed, it has a distinct effect on the radio channel which can be utilized for HAR.

In acoustic-based HAR, the system detects and recognizes human activities as well as the environment by analyzing the audio signals. Acoustic-based HAR leverages the distinct sound of each activity as a specific signature to detect and recognize human activities and events. Related research topics to the sound-based HAR can be categorized as activity and event detection and recognition, scene classification, and localization. In this work, our focus is to recognize activities that extract distinct sound signatures from the input data to recognize activities such as falling, walking, watching TV, reading, sleeping, etc.

Nevertheless, each of the abovementioned HAR methods suffer from major drawbacks, such as limited resolution of DFRAR systems in detecting fine-grained activities, and the ineffectiveness of acoustic-based HAR in detecting silent and low-volume activities, especially in noisy environments. Hence, a new solution is required for tackling these shortcomings, and in doing so, increasing the number of recognizable activities.

## 2. State of the Art

Among DFRAR systems, Wi-Fi-based methods are commonly utilized due to their available infrastructure and low employment cost [4]. In WiFall [3], the authors design a fall detection and activity recognition system using the 5 GHz frequency band of the IEEE 802.11n standard. The proposed system extracts Channel State Information (CSI) data using the 802.11n CSI Tool [5] and then uses a Local Outlier Factor (LOF)-based algorithm to detect the periods of activity by spotting these intervals as anomalies from the steady-state (intervals of no activity). The authors extract seven statistical features and then feed these features to two classifiers, namely Support Vector Machines (SVMs) and Random Forests. Their work shows an overall precision of 89% in a dormitory environment, which was lower than the accuracy acquired in the lab environment, indicating the importance of the environmental factors in recognition accuracy. In [6], an attention-based bi-directional Long Short-Term Memory (ABLSTM) network is utilized to classify six activities given only the amplitude information of the raw CSI data, resulting in 97% accuracy. In [7], the authors propose a 1D CNN architecture as a replacement for conventional classifiers and show how it matches (or even outperforms) the performance of high scoring models, given only raw, unprocessed data.

WiAct [8] is another system that tries to classify nine human activities and steady-state periods using CSI amplitude data. In WiAct, the data are preprocessed by a low-pass Butterworth filter to reduce the effect of noise. Then, an adaptive activity cutting algorithm based on signal variance is applied to detect the onset and offset of activities. After activity detection, WiAct uses a three-layer Extreme Learning Machine (ELM) network to classify the activities based on their Doppler shift correlation values and achieve an accuracy of 94.2%. Ding, J. and Wang, Y. [9] used a decision tree to detect activities based on statistical features of the CSI data. The authors used wavelet transform to denoise the data and leveraged aRecurrent Neural Network (RNN) on channel power variation and Short Time Fourier Transform (STFT) of the data to recognize and classify six activities with an accuracy of 98%.

G. Qinhua et al. [10] used multichannel CSI data to construct a radio image and showed that radio frequency (RF) image features could yield better recognition results. In [11], the authors tackle the problem of small datasets and different environmental effects on HAR using the CSI. To overcome these challenges, they extract the activity-related information from the CSI data by eliminating the environment’s static information. Then, they feed the CSI correlation features to Convolutional Neural Network (CNN) layers with bi-directional Long Short-Term Memory (LSTM) to recognize six activities. The proposed method can recognize activities with only one training sample from a new environment and one from a previously seen environment for each activity, facilitating one-shot learning and overcoming environmental dependencies. In [12], the CSI acquired from multiple access points (APs) is utilized to recognize four different activities. The classification is performed using CNN layers which are fed with heatmaps of the CSI data. The accuracy of the proposed method is evaluated with a different number of APs and people present in the environment. The results show that increasing the number of APs can improve accuracy. However, having more than one person present in the environment decreases the accuracy. In [13], the CSI phase difference acquired from commodity Wi-Fi devices is used to monitor humans’ breathing and heartbeat. The authors apply wavelet transform to separate heartbeat and breathing signals and use root-Multiple Signal Classification (MUSIC) for multiple persons’ breathing rate and a Fast Fourier Transform (FFT)-based method for heart rate estimation. TW-See [14] is another HAR system that uses CSI data to recognize human activities behind a wall. TW-See uses an Opposite Robust Principal Component Analysis (PCA) to extract features and identify six activities with an accuracy of 94.4% using an Artificial Neural Network (ANN). In [15], authors show that increasing bandwidth and using higher frequency bands could improve the accuracy and range of activities to be recognized by increasing the limiting resolution between different activities that results from using narrow-band channels. In [16], authors represent Splicer, a method to obtain an accurate power delay profile of the channel by increasing the bandwidth via using several neighboring channels, which could be useful for more accurate radio frequency (RF)-based localization tasks. In [17], authors addressed the problem of temporal phase rotation of CSI data and proposed a calibration method using a Vector Network Analyzer (VNA) to mitigate this adverse effect in order to obtain more accurate motion sensing using CSI data.

Based on the recent work in the RF-based HAR, we can conclude the following remarks [2,3,4,5,6,8,9,10,11,12,13,14,15,16,17,18,19]:

1. Most RF-based HAR systems use machine learning methods, specifically SVM and ANN, for classifying human activities based on extracted features in the frequency domain involving Fourier or wavelet transform.
2. The domain of activities that can be recognized is limited to physical activities, mainly walking, falling, sitting, bending, etc., or they have strict constraints such as low distance between the sensor and the subject, or stationary subject, etc.
3. The accuracy of activity recognition is correlated with the number of individuals and the number of sensors deployed in the environment.
4. CSI data experience temporal phase rotation, which limits their performance for HAR.

Therefore, despite the non-invasive nature and excellent performance of Wi-Fi-based methods in an ideal scenario, they suffer from two drawbacks: The range of activities that can be detected is limited or highly constrained. Second, the environment plays a crucial role in the detection rate [3], meaning that the accuracy and range of these methods are limited to the received signal strength and CSI, which are external factors in a DFRAR system. In fact, this becomes crucial when the people’s lives depend on the functionality of the device. The first drawback is due to the nature of the electromagnetic waves; nevertheless, DFRAR methods in [20,21] tried to overcome the second limitation, by using a separate device for HAR purposes instead of using the available Wi-Fi devices.

In [22], the author presents a fall detection system based on smartphone audio features. Four audio features and four classification algorithms were investigated. The results showed the best performance for ANN using spectrogram as the input feature, yielding over 98% accuracy. In [23], the authors use sparsely deployed microphones in the environment to first localize the sound event. After localization, they separate the sound event source and use a combination of CNN and convolutional LSTM to classify ten household sound events based on the Mel-spectrogram feature. Kim, Jinwoo et al. [24] investigated the classification of 12 sound events, half of which were considered emergency sound events, such as screams and explosions. The authors extracted the Log-scaled Mel-spectrogram as input features for two classification algorithms, CNN and LSTM, and demonstrated the superiority of CNN in classification over LSTM. In [25], a Residual Neural Network (ResNet) with convolutional layers is used to classify ten household activities using Log Mel-band energies and achieved an overall accuracy of 87.2%. Wireless Acoustic Sensor Network (WASN) is another system proposed by Alsina-Pagès et al. [26]. The proposed system collects and analyzes the audio data collected from several points in the home environment and categorizes fourteen events using Mel Frequency Cepstral Coefficients (MFCC) and Discrete Cosine Transform (DCT) as input features to a combination of K-means, Extended K-means, and SVM. In [27], an ensemble learning method is proposed for fall detection in an indoor environment. This method uses an ensemble of K-Nearest Neighbors (KNN), SVM, and CNN to classify actual fall and fall-like sounds. MFCC and spectrogram were the extracted features for classification. This method improves the accuracy from 87% to 94% compared to single classifiers. In [28], the authors propose a new feature for fall detection, namely, acoustic Local Ternary Patterns, and use an SVM classifier to detect falls.

One of the rich resources for audio-related research topics is the Detection and Classification of Acoustic Scenes and Events (DCASE) challenges held annually since 2016. DCASE challenges include several tasks such as acoustic scene classification and sound event localization and detection. This challenge provides a dataset and a baseline model for each task [29,30,31,32]. The proposed model in [33] was the winner of the acoustic scene classification task of the DCASE 2020 challenge. The proposed system used Mel-spectrogram and delta features of Mel-spectrogram with three parallel ResNets to improve the classification accuracy from 54% in the baseline model to 74.4%. In [34], the authors used data augmentation to increase the training dataset for the sound event localization and detection task. They became the winner of the 2020 challenge by using ensemble learning, Convolutional Recurrent Neural Networks (CRNN), and CNN as classifiers and Mel-spectrogram, Generalized Cross-Correlation (GCC), and acoustic intensity vector as input features.

Based on the recent work in the acoustic-based HAR, we can conclude the following remarks [22,23,24,25,26,27,28,29,30,31,32,33,34]:

1. Spectrogram, Mel-spectrogram, or MFCC are the most common features for acoustic-based activity recognition. This is due to the anti-noise ability of Mel-spectrogram and MFCC, and also the 2D nature of a spectrogram, which enables employing image-based classification techniques.
2. A majority of the literature use CNNs and SVMs as part of their classification module.
3. Silent activities such as sitting down or standing up are not part of the recognizable activities.
4. Noise removal is usually not discussed in acoustic-based HAR since noise (background sounds) has the same characteristics as the source signal.
5. Noise removal requires multiple microphones and involves source localization.

Each of the RF- or acoustic-based HAR techniques mentioned above has its merits and shortcomings. RF-based methods require high frequency or high bandwidth to recognize small-scale activities such as reading a book or sleeping with high accuracy, or they require high constraints such as close range or limited movement of the subject. These drawbacks prevent the current RF-based HAR system to be practical for recognizing a wide range of activities. Likewise, acoustic-based methods are unable to detect quiet activities such as breathing, sitting, etc., in noisy environments. They require a quiet environment or sophisticated source localization and denoising techniques to separate the activities’ sound signatures from the environmental sounds. These limitations prompted us to propose a hybrid method using both RF and acoustic signals to recognize human activities. We believe by employing a hybrid method, the number of activities that could be recognized increases, and they will not be limited to the constraints set by each of the individual methods.

This paper combines both RF and acoustic-based methods to detect falling, walking, sitting down, and standing up from a chair. The only hybrid method close to our proposed techniques is DeepMV [35], which uses the amplitude information of CSI and phase shift of the reflected near-ultrasound (i.e., 20 kHz) signal from the human body. However, the focus of DeepMV is the deep learning aspect of the HAR. It also uses active sound recognition by emitting near-ultrasound waves in the environment, whereas we use a passive acoustic-based HAR. To our knowledge, this is the first work to combine DFRAR and passive acoustic-based HAR for detecting human activities. Our results show the superiority of the proposed hybrid RF–acoustic HAR over each RF- and acoustic-based approach used separately. Specifically, our main contributions in this paper are:

1. Presenting a hybrid RF–acoustic approach for HAR.
2. Demonstrating the superiority of RF-acoustic data over single sensory data for HAR regardless of the classification model.
3. Presenting an environment-invariant HAR system capable of near-perfect recognition accuracy (for the considered activities).

The rest of the paper is organized as follows. In Section 3, we discuss the system design of the hybrid RF–acoustic-based HAR along with the implementation procedure. Section 4 is dedicated to results and discussion of our proposed method. Section 5 concludes this paper with the conclusion and future works.

## 3. System Design

**Figure 1:**
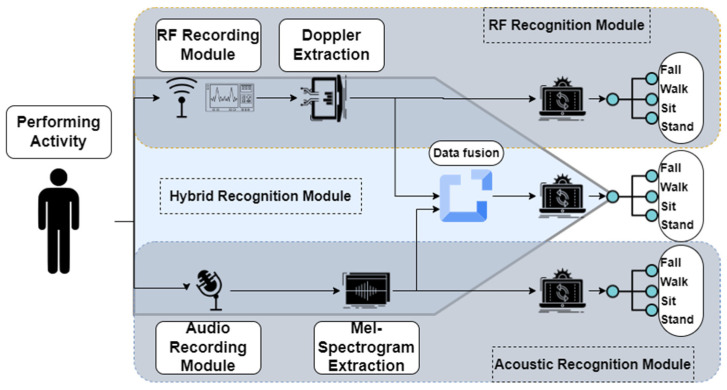
Proposed system overall design.

The overall system design of the proposed method is shown in Figure 1. We observe two types of signals from the environment, namely radio frequency signals and acoustic signals. Important features such as the Doppler shift from the RF data and Mel-spectrogram from the acoustic data are extracted. The features are then fussed using a data fusion technique and sent to activity classifiers. Each part is discussed below.

### 3.1. RF-Based Recognition Module

In the RF module, the aim is to extract the characteristics of the time-varying channel due to human activity. In this regard, RF signals with specific frequencies and phases are transmitted in the environment. Then, these signals are recorded by the receivers in the environment. The recorded signals contain information about the RF channels and their time-varying characteristics due to human activities. Analysis of these received signals can determine the type of activity being performed in the environment.

The overall steps in RF-based HAR are: (i) acquiring the CFR data, (ii) preprocessing the collected RF data, (iii) feature extraction, and (iv) activity classification using machine learning methods or mathematical modeling [18].

#### 3.1.1. Wideband Channel Measurement

Wideband channel measurements are either performed in the frequency domain or the time domain. To measure the channel in the frequency domain, stepped frequency sweeping is utilized to measure the channel at different frequency tones in the desired bandwidth. This process is performed by a Vector Network Analyzer (VNA), which extracts the complex frequency response of the channel by calculating the *S*_21_ parameter [36]. The channel transfer function is a function of the *S*_21_ parameter as [36]

(1) $$H\left(\omega \right)\propto S_{21}\left(\omega \right)$$

Considering the frequency span used in VNA as the trigger, we can say $S_{21}\left(t,f\prime \right)\propto H\left(t,f\prime \right)$, where f′ is a member of the frequency span.

#### 3.1.2. Channel Impulse Response Estimation

In Wi-Fi-based HAR methods, usually Received Signal Strength Indicator (RSSI) and CSI are utilized to gain insight into the channel and the effect of human activities [18]. However, by obtaining the channel impulse response (CIR), we can provide much more data than RSSI or CSI. Indeed, CIR can give us information pertaining to the RF channel and its transformations due to the environment and human activity. Each CIR element contains the information about the wireless propagation channel from the transmitter to the receiver [18], which can be defined as [37]

(2) $$h\left[k;\tau \right]=\sum_{m=1}^{M}c_{m}\left[k\right]\;e^{-j\left(2\pi f_{c}\tau_{m}\left[k\right]+\theta_{m}\right)}\delta \left(\tau -\tau_{m}\left[k\right]\right),\;k\in \left\{0,\;\ldots ,\;K-1\right\}$$

where *K* is the total number of samples, *M* is the number of scatterers, $c_{m}$ and $\tau_{m}$ are the corresponding amplitude and delay of the *m*-th scatterer, $f_{c}$ is the center frequency, and $\theta_{m}$ is the random initial phase. By using WB measurements, we would have a span frequency of $f^{\prime }\in \{n\left(\delta f\right)|-\left[\frac{N}{2}\right]<n<\left[\frac{N}{2}\right]\}$ where [.] is the floor function, N is the number of subcarriers, and δf is the spacing between subcarriers. In this way, we can define the channel transfer function as

(3) $$H\left[k;f\prime \right]=\sum_{m=1}^{M}c_{m}\;\left[k\right]e^{-j\left(2\pi \left(f_{c}+f^{\prime }\right)\tau_{m}\left[k\right]+\theta_{m}\right)}.$$

#### 3.1.3. Detection Problem

Assuming that all the undesired scatterers are static, and only one activity is happening in the environment, Equation (3) will be changed into

(4) $$H\left[k;f\prime \right]=c_{d}\;\left[k\right]e^{-j\left(2\pi \left(f_{c}+f^{\prime }\right)\tau_{d}\left[k\right]+\theta_{d}\right)}+\sum_{m=1}^{M}c_{m}\;e^{-j\left(2\pi \left(f_{c}+f^{\prime }\right)\tau_{m}+\theta_{m}\right)},$$

where $c_{d}\left(t\right)$ and $\tau_{d}\left(t\right)$ are the time-varying amplitude and delay of the activity we want to recognize. We define the detection problem as

(5) $$\{\begin{array}{l} {ℋ}_{0}:\;H\left[k;f\prime \right]=n\left[f\prime \right],\\ {ℋ}_{1}:\;H\left[k;f\prime \right]=s\left[k;f\prime \right]+n\left[f\prime \right], \end{array}$$

where

(6) $$\begin{array}{cc} s\left[k;f\prime \right] & =c_{d}\;\left[k\right]e^{-j\left(2\pi \left(f_{c}+f^{\prime }\right)\tau_{d}\left[k\right]+\theta_{d}\right)},\\ n\left[f^{\prime }\right] & =\sum_{m=1}^{M}c_{m}\;e^{-j\left(2\pi \left(f_{c}+f^{\prime }\right)\tau_{m}+\theta_{m}\right)}. \end{array}$$

By taking the Discrete Fourier Transform (DFT) of H[k;f′] with respect to k, we have

(7) $$\begin{array}{cc} \hat{H}\left[f;f^{\prime }\right] & =\sum_{k=0}^{K-1}s\left[k;f^{\prime }\right]e^{-j\left(2\pi f\right)\frac{k}{K}}+\sum_{k=0}^{K-1}n\left[f^{\prime }\right]e^{-j\left(2\pi f\right)\frac{k}{K}}\\ & =\sum_{k=0}^{K-1}c_{d}\;\left[k\right]e^{-j\left(2\pi \left(f_{c}+f^{\prime }\right)\tau_{d}\left[k\right]+\left(2\pi f\right)\frac{k}{K}+\theta_{d}\right)}+n\left[f^{\prime }\right]\delta \left[f\right]. \end{array}$$

#### 3.1.4. Doppler Shift Estimation

In the static environment scenario, Doppler shift is an effective technique to eliminate the effects of stationary signals [8,15,18,38,39]. In fact, during an activity, movement of the desired scatterer (e.g., human body parts) causes a Doppler shift with a distinct pattern. In this paper, we use the mean Doppler shift defined as [40]

(8) $$B\left[f\prime \right]=\frac{\sum_{f=0}^{K-1}f{\left|\hat{H}\left[f;f^{\prime }\right]\right|}^{2}}{\sum_{f=0}^{K-1}{\left|\hat{H}\left[f;f^{\prime }\right]\right|}^{2}},$$

The mean Doppler shift is fed to the activity recognition module as the RF feature for the RF data.

### 3.2. Acoustic-Based Recognition Module

In sound-based HAR, the objective is to analyze the input audio and detect the activity being performed in the environment. However, unlike RF-based techniques, the environment and the objects are not irrelevant to activity detection. Knowing the environment and distinct sound signatures can help detect the kind of activity being performed. For instance, detecting the sound of running water can indicate washing hands or washing dishes.

This module comprises two stages, representational stage and the classification stage. The aim of the representational stage is to extract the features pertaining to audio events from the raw input audio signal frames. In the classification stage, the extracted features are fed to a machine learning classification algorithm for activity recognition.

#### 3.2.1. Received Signal Model

In general, for an environment with *M* audio source and *N* receivers (microphones), the received signal model in matrix form is [23]

(9) X=Gs+z

in which $X=\left[x_{1},x_{2},\ldots ,x_{N}\right]$ is the received signal in *N* microphones, $s=\left[s_{1},s_{2},\ldots ,s_{M}\right]$ is the signals from *M* sources in the environment, *z* is the noise vector received in the microphones, and

(10) $$G=\left(\begin{array}{ccc} G_{11} & \cdots & G_{1M}\\ \vdots & \ddots & \vdots \\ G_{N1} & \cdots & G_{NM} \end{array}\right),\;G_{nm}=e^{jkr_{nm}}/r_{nm}$$

is the steering matrix, with $r_{nm}$ representing the distance between source *m* and *n*.

#### 3.2.2. Preprocessing and Feature Extraction

In the preprocessing step, we need to equalize input data samples by truncating or zero-padding, since the classification module is sensitive to the input data size. Then, we need to extract the desired feature. Features that are most useful for audio classification include spectrogram, MFCCs, GCC, and intensity vector. In this paper, we chose the Mel-spectrogram as an acoustic feature due to the following reasons:

1. The inherent nature of the Mel-spectrogram to use the Mel scale instead of the frequency scale is beneficial in classifying audio data with distinct frequency specifications based on the human auditory system.
2. We can interpret spectrograms as images and benefit from the state-of-the-art image classification techniques on our data.
3. The anti-noise ability of the Mel-spectrogram.
4. Mel-spectrogram uses a decibel scale that can illustrate the auditory data better for image-based classification techniques.
5. Since our RF features are of the spectrogram nature, using the spectrogram for the acoustic feature is beneficial for data fusion.

**Figure 2:**
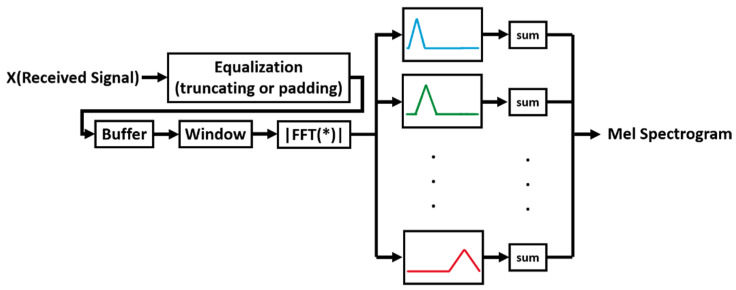
Equalizing and extracting Mel-spectrogram of the acoustic input signals. FFT(*) is the frequency-domain representation of the input * using FFT. Mel Filter Bank is represented by band-pass filters with different colors for better visibility.

Figure 2 shows the overall preprocessing and feature extraction steps.

Furthermore, because of the sensitivity of some classification algorithms to the input data scale, including SVM and ANN, it is good practice to standardize the input data, especially in our case since RF and acoustic features have different scales. Hence, to standardize our features, we remove the mean and scale the features to unit variance [41].

### 3.3. Data Fusion

**Figure 3:**
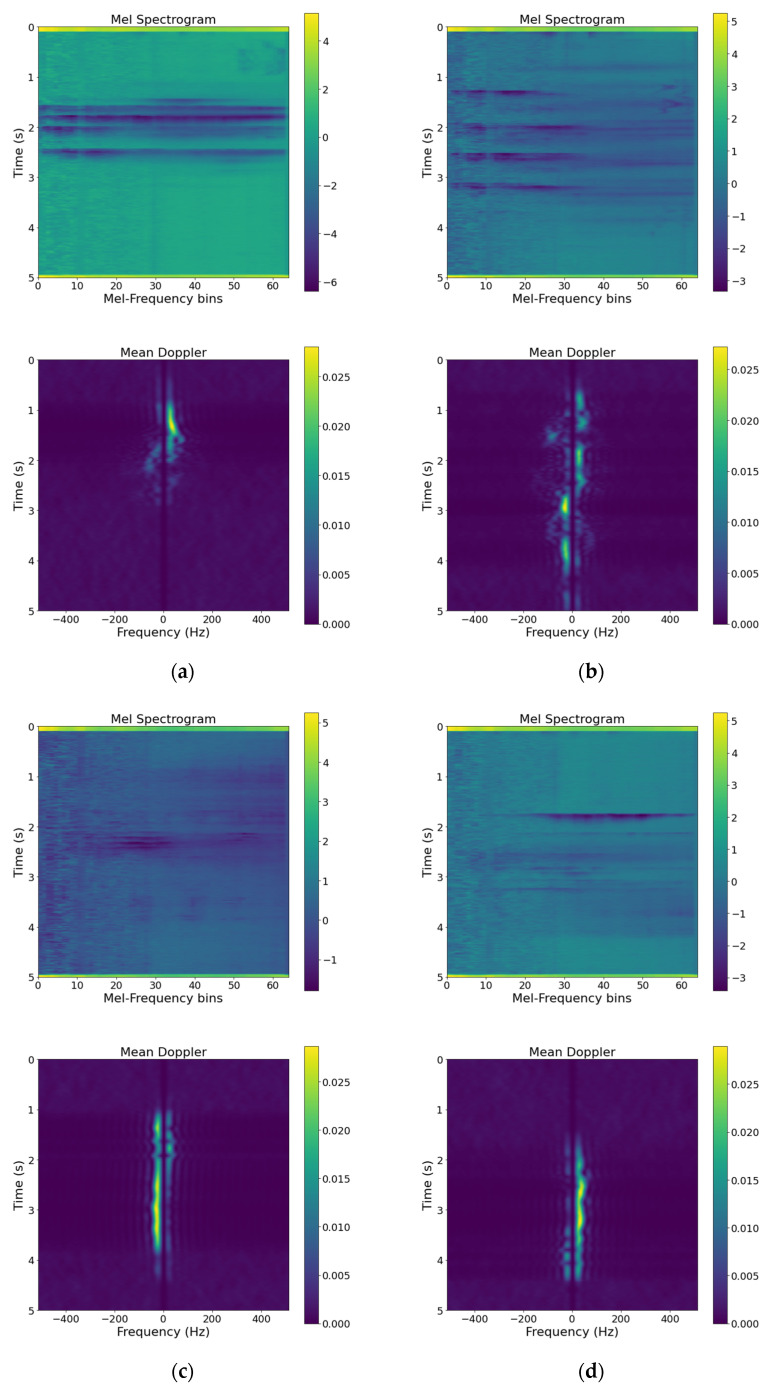
Extracted Doppler and Mel-spectrogram features from recorded data of 4 activities: (**a**) falling activity; (**b**) walking activity; (**c**) sitting activity; (**d**) standing up activity.

After extracting the Doppler shift and Mel-spectrogram features from input data and standardization, we need to fuse the input data. Fusion methods fall under 3 categories: data-level fusion, feature-level fusion, and decision-level fusion [19]. Since our sensory data are of a heterogeneous nature, we require a feature-level fusion for combining our sensory data. Feature aggregation, which is the process of concatenating extracted features, is in the category of feature-level fusion techniques. However, this procedure will result in a high-dimensional feature vector in orders of hundred thousand. Figure 3 illustrates samples of extracted features from input data for the 4 recorded activities.

We can derive 2 points from Figure 3:

- As mentioned earlier, the scale of the acoustic and RF features is different.
- Extracted features have sparse data.

Since sparse features can increase the complexity of the classification models, and cause additional problems such as overfitting, we need to further process these features and make the features denser. As a result, after flattening and aggregating the multi-dimensional features, we apply PCA [41,42] and select from the most important components, in our case 50 components. By applying PCA, in addition to reducing the sparse feature space, we further fuse the features.

### 3.4. Classification

To demonstrate the increased performance due to RF–acoustic fusion regardless of the classification technique, we include 6 classifiers in this work. We implemented the following classifiers for our proposed approach: (i) Multi-Layer Perceptron (MLP), (ii) SVM, (iii) Random Forest, (iv) Extremely Randomized Trees (ERT), (v) K-Nearest Neighbors (KNN), and (vi) Gradient Tree Boosting (GTB).

#### 3.4.1. MLP

Given the dense and highly processed nature of input data, despite the superior performance of more complicated models such as CNN, to avoid overfitting, MLP seems a more appropriate choice for our data. We chose 2 layers with 100 neurons, ReLu activation, and adam optimizer for our MLP to evaluate the classification performance of neural networks on our data [41,43].

#### 3.4.2. SVM

SVM is one of the well-known kernel method classification algorithms [44,45]. SVM aims to find a boundary among different classes by transferring the features to higher dimensions. SVMs perform well on high-dimensional data and are adaptable for different tasks because of their different kernel functions [41]. In this work, we used an SVM with Radial Basis Function (RBF) kernel, degree of 3, random state of zero, and default parameters stated in [41].

#### 3.4.3. Random Forest

Random Forest is among the ensemble learning methods. In Random Forests, the output decision is the average of an ensemble of weak classifiers (randomized decision trees) which are defined based on the training set of data. Including randomization in the structure of this classifier improves its performance by avoiding overfitting [41,46]. We used the Random Forest classifier in [41] with default parameters and random state of zero, with bootstrapping set to false.

#### 3.4.4. ERT

In ERT, the randomness of the classifier structure increases by generating a random threshold for each feature and choosing the best threshold for the splitting rule. This technique further prevents overfitting of the model [41,47]. For this classifier, we use the default parameters in [41] with bootstrapping set to false and random state equal to zero.

#### 3.4.5. KNN

In the KNN algorithm, the model classifies the input based on K-Nearest Neighbors close to the input data [41,48]. We used the default parameters set in [41] for this classifier.

#### 3.4.6. GTB

GTB belongs to the group of ensemble classifiers. However, unlike Random Forest, GTB is an ensemble of weak learners, *h_m_*, and the goal is to minimize a defined loss function by adding newer weak learners to the ensemble as in (10). For classification, a SoftMax function is applied to obtain the probability of each class prediction [49].

(11) $$\begin{array}{c} \hat{y_{i}}=F_{M}\left(x_{i}\right)=\sum_{1}^{M}h_{m}\left(x_{i}\right)\\ F_{m}\left(x\right)=F_{m-1}\left(x\right)+h_{m}\left(x\right) \end{array}$$

For the GTB, we used a validation fraction of 0.2, with early stopping with tolerance of 1 × 10^−3^ for 10 iterations. We implemented all the classifiers using the Scikit-Learn python package.

### 3.5. Implementation

**Figure 4:**
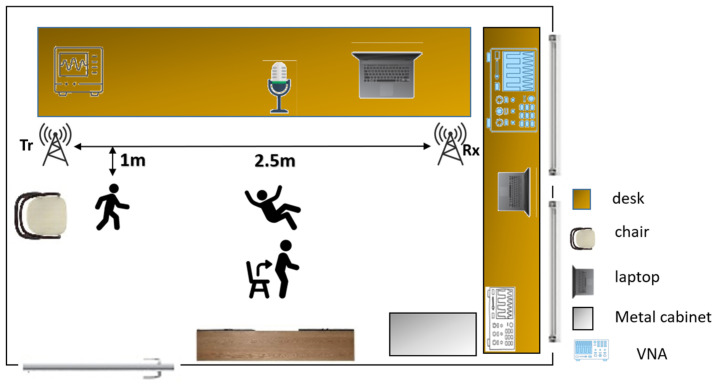
Schematic of the data recording environment. The activities of walking and falling are performed with 1 m distance to the path connecting the 2 antennas. Walking is performed from one antenna to another, parallel to the line-of-sight. The falling activity is performed in the middle section without blocking (or disturbing) the line-of-sight between the antennas, and in different falling angles. The sitting and standing activities are performed on a chair which is placed in front of the microphone, between the antennas, without blocking the line-of-sight path.

Figure 4 shows a schematic of our measurement setup in the lab environment. We did not modify the environment to keep the scene as close to the real-world environment as possible. As shown in Figure 3, the Omni-directional antennas are 2.5 m apart, and the microphone array is placed in the middle of the room on the table. We used two separate Windows-operated laptops to record RF and acoustic data simultaneously, synced with the computers’ online clock. We performed our approach in 4 steps as recording module setup, performing selective activities, recording the RF–acoustic hybrid dataset, and activity recognition module.

#### 3.5.1. Recording Module Setup

CSI tools, VNAs, and software-defined radio (SDR) platforms can provide us with CFR. Here, we chose VNA because it can provide us with complete RF channel information, and unlike the CSI tool, it is more robust to noise and phase temporal rotation [17]. Furthermore, VNAs are more configurable in comparison with other RF recording devices and are not acquainted with the Wi-Fi sensing (CSI) challenges such as “Coexistence of Wi-Fi Sensing and Networking” [18].

To make a fair comparison with Wi-Fi-based HAR devices, we abided by the IEEE 802.11n standard. Specifically, we used the lowest grouping of the IEEE 802.11n standard to collect 58 subcarrier data points in the 40 MHz channel bandwidth. We chose the wider 40 MHz bandwidth over the 20 MHz band because it can provide us with richer information about the environment [15].

One difference between our RF setup and the setup provided by the Wi-Fi-based devices is the more accurate phase shift response. Based on the findings of [3], we chose one meter as the height of our antennas. To obtain the maximum sampling rate of the VNA without introducing too much noise in the data, we set the Intermediate Frequency (IF) of the VNA to 100 kHz. This setup gives us a sampling rate of 110 samples per second, which is fast enough to detect the targeted activities even though it is lower than the highest sampling rate provided by the CSI tool (1000 sample/s and higher). This means that the computation load of the system will be lower.

For the acoustic data collection, we chose the Zylia ZM-1 spherical microphone array. This microphone provides us with 19 Micro-electromechanical system (MEM) microphones and can provide richer content with 19 channels in comparison with single microphones. We chose the usual sampling rate of COTS microphones, which is 48 kHz. In addition, this microphone is compatible with MATLAB software, which enables us to have a synchronized data collection session with the VNA, operated remotely with the MATLAB software. For the Mel-spectrum, we utilized ‘Hann’ window in our case.

#### 3.5.2. Selected Activities

To show the performance increase due to RF–acoustic data fusion, we chose walking, falling, sitting, and standing as our activities. We choose walking and falling because audio-based HAR models perform well on these activities, and unlike RF-based HAR, they are more robust to walking and falling pattern and location changes. Sitting and standing activities were chosen because RF-based HAR models perform well on recognizing these activities. However, audio-based HAR systems do not perform well on these activities because of their silent nature, and they are usually not included in audio-based HAR literature. These 4 activities were performed in the Lab environment as described below:

- Walking: Walking activities were performed parallel to the line-of-sight (LOS) with a 1 m distance from the LOS. Furthermore, 40 samples of walking activity by one subject were performed without any evident pattern (i.e., walking randomly in the room) for evaluation purposes.
- Falling: Falling activities were performed between the antennas in 4 different directions, falling toward the receiver with a zero degree angle, toward the receiver with a 45 degree angle, and likewise toward the transmitter.
- Sitting: Sitting activities were performed on two different chairs between the antennas. In this activity, the subjects were asked to sit on the chair from a standing position during each recording session.
- Standing: Standing activities were performed the same way as the sitting activity. The subjects were asked to stand up from the chair during the recording sessions.

#### 3.5.3. Collected Dataset

We asked 4 participants to perform four activities, including falling, walking, standing, and sitting on a chair. Each activity was recorded in a 5 s period to avoid spotting activity intervals (activity detection) and focusing on activity recognition. Table 1 summarizes the number of activities performed by four persons. Furthermore, we recorded 40 data samples while no one was present in the room as steady-state data.

#### 3.5.4. Activity Recognition Module

In this module, we trained the 6 classifiers in Section 3.4 using 80% of the data collected by Male 1, Male 2, Female 1, and all the steady-state data as the training set (excluding waking randomly) and allocated the rest as the test set. Furthermore, to evaluate the performance and robustness of the trained models on data collected from a new person, and the effect of pattern-less walking, we fed the data of Male 3 (unseen set) and the walking randomly of Male 1 (random set) to the classifiers, respectively. The results obtained from the trained models are presented in the following section.

## 4. Results and Discussion

To compare the effect of hybrid data, we trained our classifiers using hybrid data, RF data, and acoustic data. We trained and evaluated the network with 50 components extracted from Mel-spectrogram and Doppler features after applying PCA. Our evaluation metric is the overall accuracy. Nevertheless, other metrics such as precision and recall can be calculated using the provided confusion matrixes for the unseen set, but they are omitted here for brevity.

### 4.1. RF-Based HAR

**Figure 5:**
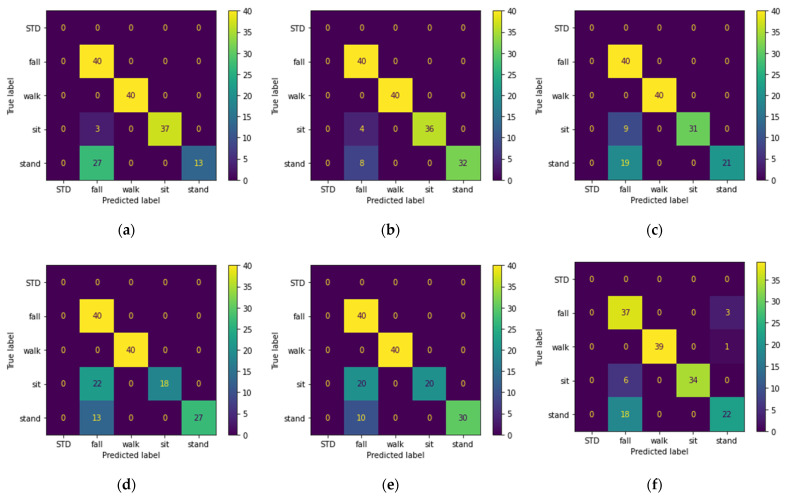
Confusion matrix of the activities in the unseen dataset predicted using: (**a**) MLP; (**b**) SVM; (**c**) Random Forest; (**d**) ERT; (**e**) KNN; (**f**) GTB; classifiers trained with top PCA components extracted from Doppler shift feature of the train dataset.

Table 2 shows the results obtained from training the models with top 50 PCA components extracted from Doppler features. Confusion matrixes of all models on the unseen set is shown in Figure 5.

### 4.2. Acoustic-Based HAR

**Figure 6:**
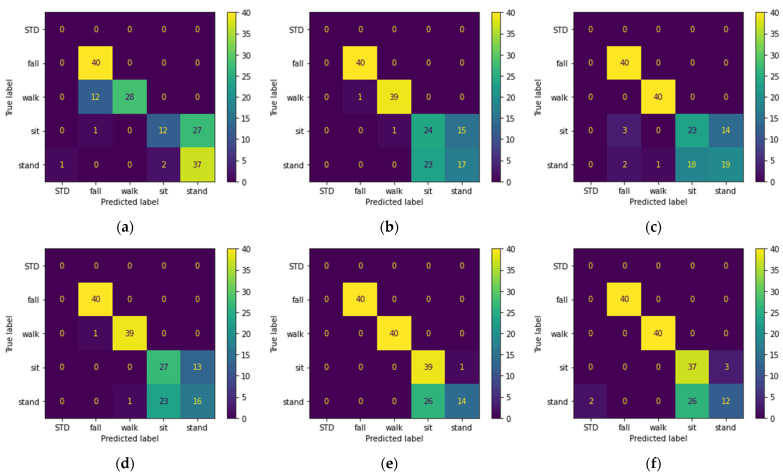
Confusion matrix of the activities in the unseen dataset predicted using: (**a**) MLP; (**b**) SVM; (**c**) Random Forest; (**d**) ERT; (**e**) KNN; (**f**) GTB; classifiers trained with top PCA components extracted from Mel-spectrogram features of the train dataset.

Table 3 shows the results obtained from training the models with top 50 PCA components extracted from Mel-spectrogram features. Confusion matrixes of all models on the unseen set is shown in Figure 6.

### 4.3. Hybrid RF–Acoustic-Based HAR

**Figure 7:**
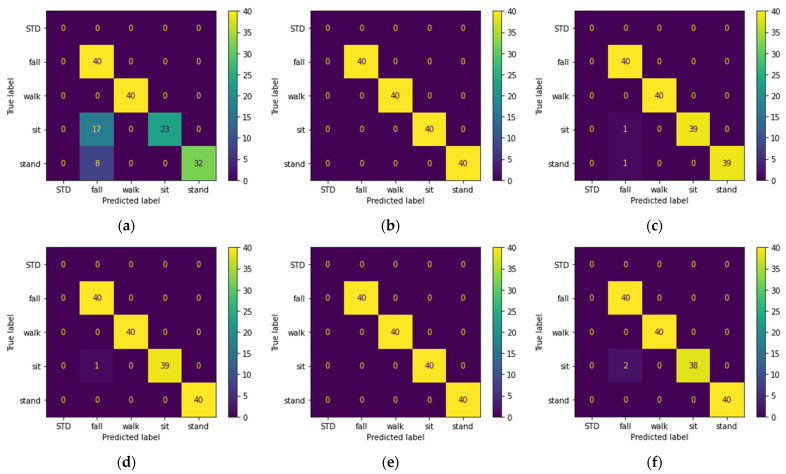
Confusion matrix of the activities in the unseen dataset predicted using: (**a**) MLP; (**b**) SVM; (**c**) Random Forest; (**d**) ERT; (**e**) KNN; (**f**) GTB; classifiers trained with top PCA components extracted from hybrid Doppler–Mel-spectrogram features of the train dataset.

As mentioned before, for hybrid data, we used aggregation to fuse Doppler and Mel-spectrogram features before applying PCA. The results are shown in Table 4 and the confusion matrixes of all models on the unseen set are shown in Figure 7.

### 4.4. Discussion

Since the test set and training set are complementary subsets of the activities performed by Male 1 and Male 2 and Female 1 (excluding waking randomly), they are expected to yield better results, as is seen in the previous section. This is why we consider the performance of the models on the unseen and random sets as our evaluation criteria, since these sets can better represent the real-life scenarios. Based on the classification results, we can conclude the following statements:

1. Looking at Table 2, we observe that three of the shallow models were able to recognize the pattern-less (random set) walking activity. We believe this is due to applying PCA on the input data before feeding them to the model. Figure 5 shows that five of the six models correctly classified the falling and walking activities. However, the main drawback of all the models is the misclassification of the sitting and standing activities as falls, and the best performance belongs to the SVM classifier.
2. Table 3 shows that audio features are more robust to pattern changes of the walking activity, and all the models except MLP achieve 100% accuracy on the random set. However, as seen in Table 3 and Figure 6, the performance of the models on the unseen set using acoustic features is lower than that of the RF features. The lower performance of the models is mainly due to misclassification of the sitting and standing activities with each other, which was expected given the silent nature of these activities. Looking at Figure 5 and Figure 6 and disregarding the MLP, we can see that audio features perform better on walking and falling activities in terms of precision and recall. For acoustic features, despite lower accuracy of KNN for the test set, KNN outperforms the rest of the classifiers.
3. The results obtained from hybrid data classification, Table 4 and Figure 7, show that the hybrid method matches or outperforms the state of the art, and we can see the increased performance for all models. By fusing RF and acoustic data, the problem of false positives in the falling activity with RF-based classification and misclassification of the silent activities in acoustic-based classification is solved. In other words, RF and acoustic features can complement each other; the RF feature can recognize silent activities, which audio features lack, and audio features are more invariant to environment setup, activity pattern, and subject location, in comparison with RF features. Figure 7 shows that the SVM and KNN classifiers perform better than ERT, Random Forest, and GTB by a close margin.

#### 4.4.1. Acoustic Noise Effects

**Figure 8:**
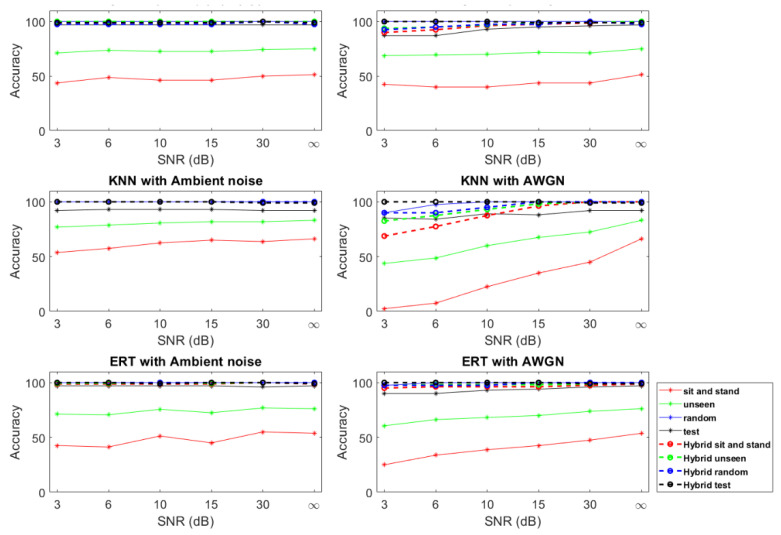
Effect of ambient noise and AWGN on performance of the acoustic-based and hybrid-based HAR system. Different SNR levels are included for each kind of noise. Accuracy of acoustic and hybrid systems with SVM, KNN, and ERT are shown. Dashed lines correspond to the performance of the hybrid system. Sit and stand, unseen, random, and test refer to the corresponding datasets used for evaluation.

As mentioned in previous sections, acoustic-based HAR systems are unable to detect silent activities, especially in noisy environments. We thus separately added ambient noise recorded in the lab environment and additive white gaussian noise (AWGN) to examine the effect of noise on acoustic-based as well as on the proposed hybrid-based systems. To do so, we added ambient noise and AWGN with different signal-to-noise ratio (SNR) levels to all the acoustic raw data and evaluated the systems on three evaluation datasets. Furthermore, to show the effect of noise on silent activities (sitting and standing up), we recorded the accuracy of the models for the sitting and standing up activities of the unseen dataset. Figure 8 shows the accuracy of the three top performing classifiers on the unseen dataset from the previous section for different levels of the SNR.

Based on the results from Figure 8, we can see that in every case, the hybrid-based system outperforms the acoustic-based system. Furthermore, we can observe that the ERT and SVM classifiers are more robust to noise and have better performance than the KNN classifier in the presence of AWGN. In addition, we can see that the acoustic-based system performance on sitting and standing up activities is lower for all the classifiers, and these activities, because of their silent nature, are more prone to noise, as indicated in the introduction section. However, we can see that by employing a hybrid approach, we can counteract the unwanted effect of noise on our system’s performance.

#### 4.4.2. Sensor Setup and Environmental Effects

**Figure 9:**
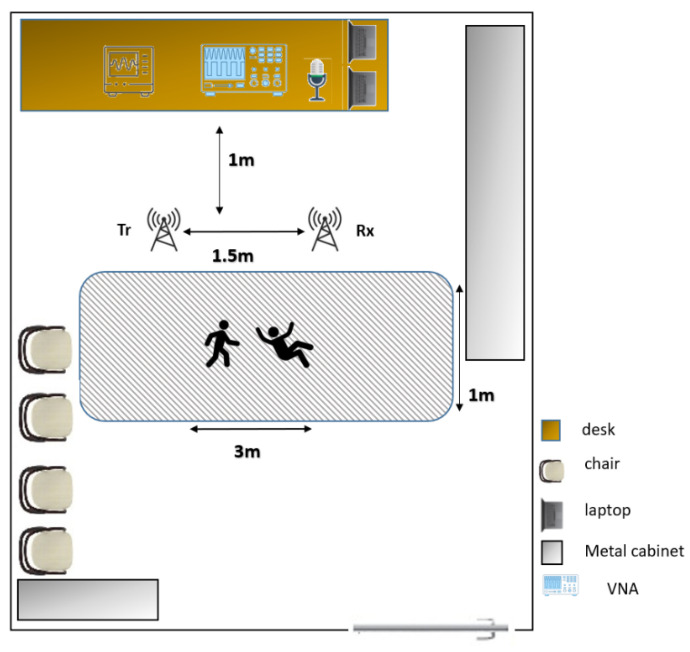
Schematic of the new room data recording setup. Walking and falling activities were performed in the dashed area without blocking the line-of-sight path between Tr and Rx. The walking activity was performed without any pattern, similar to the walking activity in the random set. The falling was performed in different directions in the dashed area. Measurement device placements in this room were different from that of the lab environment.

To analyze the effect of the environment and the RF sensor placement, and further distinguish among different classifiers, we recorded 10 falling and 60 walking samples for Male 1 in a new room with more acoustic reverberation. In the new room, the walking activity was performed without a specific pattern and the falling activity was performed like the falling activity in the lab. The schematics of the room and sensor placements are shown in Figure 9.

We evaluated the recorded data in the new room with all the models. Table 5 shows the precision, recall, and overall accuracy of the models evaluated by recorded data in the new room.

Looking at Table 5 we can see that KNN, ERT, and GTB classifiers are more robust to environment change and sensor setup given hybrid data and KNN, MLP, and GTB yield 100% accuracy for acoustic data. Furthermore, none of the classifiers achieve 100% accuracy for RF data.

Overall, the ERT classifier is able to achieve 100% accuracy for the random and new room dataset. Given its robustness against AWGN compared to KNN, we can say that ERT outperforms the rest of the classifiers.

One of the existing challenges in HAR is to increase the range of recognizable activities. Previous works in RF-based HAR suggest using higher frequency bands with larger bandwidths to increase the range of recognizable activities, including fine-grained activities such as typing. However, these solutions have their own constraints given the limited available bandwidth in lower frequency bands, and higher power loss and noise contamination in higher frequency bands. Seeing how fusing RF and acoustic features enabled the recognition of silent activities, which were recognized poorly by acoustic-based models, and increased precision and recall of falling and walking activities compared to RF-based models, we believe RF–acoustic fusion can be an answer for expanding the range of recognizable activities by means of non-invasive sensors.

## 5. Conclusions

In this paper, we presented a hybrid RF–acoustic HAR system. We chose RF and acoustic sensors because of their non-invasive nature, privacy preservation, and lack of need for on-body sensors. We configured our RF module with IEEE standards to have a fair comparison with existing CSI-based HAR systems and make our model more adaptable for COTS devices. In doing so, given the available RF and acoustic infrastructure in every household, we believe our proposed system has the potential for easy implementation and commercialization.

By training the models using RF data, we demonstrated some limitations of RF-based HAR. Since we extracted Doppler features from RF data and mitigated the effect of the environment, the low overall accuracies achieved by RF features in Table 5 show the dependency of the model performance on sensor setup. In addition, the misclassification of siting and standing activities with falling points to the other limitation of RF-based methods in identifying activities with similar RF signatures. To overcome this problem, using higher frequency bands with larger bandwidths is suggested in the literature.

To show the advantages and disadvantages of acoustic-based HAR, we also trained our models with Mel-spectrogram features. As seen in Figure 6, acoustic features are more suited for falling and walking activities, whereas they do not perform well on silent activities, especially in the presence of noise. Therefore, overall when an activity has a distinct sound signature, acoustic-based HAR can outperform the RF-based HAR. Nevertheless, the other drawback of acoustic-based HAR is performance degradation due to reverberation and background noise. Figure 8 shows how acoustic-based systems are prone to noise in comparison with hybrid systems. Furthermore, looking at Table 5, we see how room reverberation lowered the performance of SVM, Random Forest, ERT, and GTB compared to their performance in Table 3.

In this work, given the benefits and drawbacks of RF-based and acoustic-based HAR, we proposed a hybrid RF–acoustic-based HAR. For data fusion, given the heterogenous nature of the input data, we used a feature-level aggregation data fusion technique followed by PCA to counter the sparse nature of input data (Figure 3). The proposed hybrid system showed performance improvement regardless of the classification model. For evaluating the performance of the models, in addition to using the common test set, we employed three additional sets (random, unseen, new room) with distinct differences from the train and test set. By comparing the models’ performance on the unseen set (Figure 5, Figure 6 and Figure 7), we showed that none of the models are able to achieve over 92.5% recognition accuracy with only one type of sensor. By analyzing the results obtained from the effect of acoustic noise (in Figure 8) and the new room dataset (in Table 5), we showed how ERT outperforms the rest of the models and is more robust to noise, environmental factors, and sensor setup.

Finally, analyzing the performance of the models for the falling, sitting down, and standing up activities revealed how RF and acoustic signals can complement each other. Based on this observation, we believe using RF–acoustic data fusion could increase the range of recognizable activities in addition to increased performance. RF-based HAR systems can recognize activities that involve motion; however, they are unable to detect motionless activities such as talking on the phone, speaking, watching TV, etc., and alarming events such as explosions, screams, gunshots, etc. The same stands for acoustic-based activities in recognizing silent activities such as sitting, standing, punching, etc. Therefore, by using a fusion of these two sensory data types, we can increase the set of recognizable activities and events, as we showed for the sitting and standing activity in this paper.

For future work, we plan to include more activities and events, especially activities which are poorly recognized by RF features. In addition, the effect of the background acoustic noise on the performance of the models and countering it by adding source-localization-based denoising techniques will be analyzed. Other directions for future work could also involve the analysis of human subject localization with RF–acoustic data fusion.

## Figures and Tables

**Table 1:** Summary of the number of activities performed by the participants.

Subject	Walking	Falling	Standing	Sitting	Walking Randomly	Steady-State
Male 1	80	40	80	80	40	
Female 1	40	50	40	40	-	
Male 2	40	15	-	-	-	
Male 3	40	40	40	40	-	
Total	200	145	160	160	40	40

**Table 2:** Accuracy of classification models for RF features.

Model	Test Set Acc	Unseen Set Acc	Random Set Acc
MLP	1.0	0.812	0.95
SVM	1.0	0.925	1.0
Random Forest	1.0	0.825	1.0
ERT	1.0	0.781	1.0
KNN	1.0	0.812	0.875
GTB	0.99	0.825	0.875

**Table 3:** Accuracy of classification models for Acoustic features.

Model	Test Set Acc	Unseen Set Acc	Random Set Acc
MLP	0.91	0.731	0.925
SVM	0.97	0.75	1.0
Random Forest	0.97	0.762	1.0
ERT	0.97	0.762	1.0
KNN	0.92	0.831	1.0
GTB	0.94	0.806	1.0

**Table 4:** Accuracy of classification models for hybrid RF–acoustic features.

Model	Test Set Acc	Unseen Set Acc	Random Set Acc
MLP	0.99	0.843	1.0
SVM	0.99	1.0	0.975
Random Forest	1.0	0.987	1.0
ERT	0.99	0.993	1.0
KNN	0.99	1.0	1.0
GTB	1.0	0.987	1.0

**Table 5:** Precision, recall, and overall accuracy of models for recorded data in the new room (Hyb: hybrid data; RF: RF data; Aco: acoustic data).

Model	Fall Precision			Fall Recall			Walking Precision			Walking Recall			Overall Accuracy		
	Hyb	RF	Aco	Hyb	RF	Aco	Hyb	RF	Aco	Hyb	RF	Aco	Hyb	RF	Aco
MLP	1.0	1.0	1.0	0.16	0.3	1.0	0.12	0.58	1.0	1.0	1.0	1.0	0.24	0.64	1.0
SVM	1.0	1.0	1.0	0.23	0.29	0.14	0.43	0.55	0	1.0	1.0	0	0.51	0.61	0.14
Random Forest	1.0	1.0	1.0	0.45	0.3	0.2	0.8	0.58	0.32	1.0	1.0	1.0	0.83	0.64	0.41
ERT	1.0	1.0	1.0	1.0	0.33	0.25	1.0	0.63	0.5	1.0	1.0	1.0	1.0	0.69	0.57
KNN	1.0	0.7	1.0	1.0	0.21	1.0	1.0	0.45	1.0	1.0	1.0	1.0	1.0	0.49	1.0
GTB	0.9	1.0	1.0	1.0	0.24	0.71	1.0	0.45	0.93	0.98	1.0	1.0	0.99	0.53	0.94
